# Differential Analysis of Serum Principal Components Treated with Compound *Sophora* Decoction and Related Compounds Based on High-Resolution Mass Spectrometry (HRMS)

**DOI:** 10.1155/2020/7518479

**Published:** 2020-09-30

**Authors:** Wanjin Sun, Junjie Zhang, Conghui Zhou, Bin Yan, Quan Cai, Hongxia He, Xueyun Duan, Heng Fan

**Affiliations:** ^1^Department of Pharmacy, Hubei Provincial Hospital of Traditional Chinese Medicine, Wuhan 430061, China; ^2^Department of Pharmacy, Hubei Province Academy of Traditional Chinese Medicine, Wuhan 430074, China; ^3^Graduate School, Tianjin University of Traditional Chinese Medicine, Tianjin 300000, China; ^4^Department of Integrated Traditional Chinese and Western Medicine, Union Hospital, Tongji Medical College, Huazhong University of Science and Technology, Wuhan 430022, China

## Abstract

**Objective:**

To compare the differences in the serum principal components in ulcerative colitis- (UC-) induced rats, treated with compound *Sophora* decoction, matrine, oxymatrine monomer mixture, and indirubin monomer, and to provide a modern scientific basis for elucidating the clinical efficacy of compound *Sophora* decoction for the treatment of UC.

**Methods:**

The serum samples of rats from each group were obtained after drug administration, and the serum principal components of each group were analyzed by high-resolution mass spectrometry. Agilent Eclipse XDB C18 chromatographic column (100 mm × 2.1 mm, 3.5 m) was used for separation. The mobile phase was water (A) and methanol (B) (0.1% formic acid) gradient elution, 0–3 min (B: 20%–40%), 3–10 min (B: 40%–54%), 10–25 min (B: 54%), 25–35 min (B: 54%–70%), 35–45 min (B: 70%–80%), 45–50 min (B: 80%), 50–60 min (B: 80%–100%), 70–72 min (B: 100%–20%), and 72–77 min (B: 20%); flow rate, 300 *μ*L/min; column temperature, 40°C; and injection volume, 10 *μ*L. ESI source was selected and scanned in the positive and negative ion modes. The scanning range was 70–1500 *m*/z; ion-source gas 1 (GS1): 55 psi; ion-source gas 2 (GS2): 60 psi; CUR: 30 psi; ion-source temperature (TEM): 550°C; ion-source voltage (ISVF) : 5500 V/−4500 V; decluster voltage (DP): 100 V; collision energy (CE): 35 V/−35 V; collision energy gain (CES) : 15 V/−15 V; and data acquisition mode: IDA. After the data from each group were imported into MarkView 1.3, the molecular weights and retention times of different substances were obtained and qualitatively analyzed by ChemSpider and PeakView 2.0.

**Results:**

In the negative ion mode, 26 differential compounds were identified in the compound *Sophora* decoction group (FFKST) compared to the model group (M), and 18 differential compounds were identified in the matrine and oxymatrine group (KST) compared to the model group (M). In the positive ion mode, 11 and 7 differential compounds were identified in the compound *Sophora* decoction group (FFKST) and the matrine and oxymatrine group (KST) compared to the model group (M), respectively. The responses of all compounds in each group were compared with each other. As the different principal component substances in the indirubin group (DYH) displayed little correlation with other groups, the different components in this group were not researched thoroughly.

**Conclusion:**

By comparing the differences in the serum principal components from each administration group, we found that the FFKST group exhibited enhanced synthesis of the serum principal components; however, the compound doses of matrine and oxymatrine monomers did not exhibit the same changes in the serum principal components of UC-induced rats. Finally, the traditional Chinese medicine compound is more advantageous than monomers.

## 1. Introduction

Ulcerative colitis (UC) is a recurrent inflammatory bowel disease, characterized by chronic inflammation and ulcer formation in the colonic mucosa. The disease manifests pus and blood. The lesions are located mostly in the sigmoid colon and rectum, but they can spread to the descending colon, even affecting the entire colon. The disease is long-lasting and recurrent [1, 2]. The compound *Sophora* decoction is one of the Chinese herbal medicines, which has exhibited remarkable curative effects in the clinical treatment of UC. It consists of *Sophora flavescens* Ait., *Sanguisorba officinalis* L., *Indigo naturalis*, *Bletilla striata* Diels., and *Glycyrrhiza uralensis* Fisch. In the preliminary results, matrine and oxymatrine displayed significant anti-inflammatory activities. They reduced intestinal mucosal inflammation in experimentally induced colitis by regulating NOD2/NF-*κ*B and *β*2AR-*β*-arrestin-2-NF-*κ*B signal transduction pathways (important components to evaluate the polyjuice potion) [3, 4]. Simultaneously, indirubin promoted the expression of claudin-1, occludin, zo-1, and jam-1, suggesting that indirubin is the main effective component for UC treatment [5]. Chinese traditional medicine functions in a “multicomponent and multitarget” pathway for disease treatment by the synergistic action of its components [6, 7]. The effect of a compound or monomer depends upon its pharmacodynamic and pharmacokinetic characteristics. Therefore, by studying the differences in the main serum components, we can confirm the effectiveness of drugs and speculate about the treatment mechanism of these drugs. Based on the high-resolution mass spectrometric analysis, this experiment compared the differences in the serum principal components among the compound *Sophora* decoction, mixture of matrine, oxymatrine monomer, and indirubicone monomer and provided a modern scientific basis for elucidating the clinical efficacy of compound *Sophora* decoction.

## 2. Materials and Methods

### 2.1. Instruments

In this study, we used Shimadzu Prominence UPLC system LC-20 (Shimadzu, Japan), AB Sciex Triple QTOF 5600+ high-resolution mass spectrometer (AB Sciex, USA), BT25S 100,000th analytical balance (Saidoris, Switzerland), XW-80A vortex mixer (Jintan Yichen, China), KQ-500VD ultrasonic cleaner (Kunshan, China), TGL-16 desktop high-speed refrigerated centrifuge (Xiangyi, China), and UPT-II-10T ultrapure water machine (UPT, China).

### 2.2. Materials

*Sophora flavescens* Ait., *Sanguisorba officinalis* L., *Bletilla striata* Diels., *Glycyrrhiza uralensis* Fisch, and *Indigo naturalis* were authenticated by professor Keli Chen of the Hubei University of Traditional Chinese Medicine (Wuhan, China), and they complied with the provisions of the 2015 edition of the Chinese pharmacopeia. The reserved samples were stored in the herbarium of the Hubei Institute of Traditional Chinese Medicine (Wuhan, China); the voucher specimen numbers of *Sophora flavescens* Ait., *Sanguisorba officinalis* L., *Bletilla striata* Diels., *Glycyrrhiza uralensis* Fisch, and *Indigo naturalis* samples were 201809012, 201809004, 201809015, 201809020, and 201809009, respectively. The standard reference compounds, including indirubin (batch number: 110717-200204), matrine (batch number: 110805-200508), and oxymatrine (batch number: 110780-201508), were purchased from China Food and Drug Identification Institute (Beijing, China); 5% 2,4,6-trinitrobenzene sulfonic acid (TNBS, 3LBD6811V) was purchased from Sigma. Mesalazine granules (batch number: 170904) were purchased from the France Aifa Pharmaceutical group.

### 2.3. Experimental Animals and Treatment

A total of 28 Wistar rats (male, weight 190–210 g) provided by the Beijing Weitong Lever Laboratory Animal Technology Co. (Beijing, China, License no. SCXK (Beijing) 2016-0006) were lodged under speciﬁc pathogen-free (SPF) conditions with free access to the autoclaved food and water in the experimental animal center of the Hubei Provincial Hospital of Traditional Chinese Medicine (HPHTCM, Wuhan, China). The animals were housed under the standard laboratory conditions at a temperature of 25 ± 2°C, the relative humidity of 50–55%, and 12 h light/dark cycle and acclimatized to the environment for at least one week before animal experiments. The animal welfare and experimental procedures strictly complied with the guidelines of the Animal Research Institute Committee of HPHTCM (no. HBZYY-2018-043) and National Institutes of Health guidelines and regulations.

### 2.4. Preparation of Compound *Sophora* Decoction (FFKST)

The compound *Sophora* decoction consists of *Sophora flavescens* Ait., *Sanguisorba officinalis* L., *Bletilla striata* Diels., *Glycyrrhiza uralensis* Fisch, and *Indigo naturalis* (3 : 3:2 : 2 : 1). These herbs were soaked with water for 30 min and then boiled them twice for 1 h each time. Then, the supernatant was concentrated to 1.25 g/mL. Before using, it was diluted with saline to the dosage volume of 1 mL.

### 2.5. Preparation of Matrine and Oxymatrine Intragastric Solution

According to the original prescription, a mixture of matrine and oxymatrine (3 : 1) was used to prepare the intragastric solution [8]. Before using, it was diluted with normal saline to the dosage volume of 1 mL.

### 2.6. Grouping and Modeling of Animals

After 24 hours of fasting, the rats were anesthetized with 1% pentobarbital sodium (40 mg/kg), and then, 3 rats were randomly selected as the normal group. The remaining rats were induced to UC by administering with TNBS (5% TNBS and 50% ethanol solution were mixed in a proportion of 12 : 5, and the mixture (4.25 ml/kg) was injected into the rat colon through the anus with a rubber hose of 2 mm in diameter), and the normal group was injected into the colon with normal saline (4.25 ml/kg). Then, the rats were laid flat and provided with a free diet after they woke up naturally. When the rats developed loose stools, then the mucus and bloody stools appeared, accompanied by the loss of appetite, laziness, weight loss, arched back, and dull hair, indicating that the model was successfully induced [9, 10]. The UC-induced rats were randomly divided into the model group (M), positive control group (MS), compound *Sophora* decoction group (FFKST), matrine and oxymatrine group (KST), and indirubin group (DYH) with 5 rats in each group.

### 2.7. Drug Administration and Sampling

The positive control group (MS) was administered with 0.5 g/kg/d of mesalazine; the compound *Sophora* decoction group (FFKST) was administered with 5 g/kg/d of the crude drug; the matrine and oxymatrine group (KST) was administered with 10.83 mg/kg/d of matrine and 3.61 mg/kg/d of oxymatrine solution [11] by gavage; the indirubin group (DYH) was administered with 14.44 mg/kg/d of indirubin [12] solution by gavage. The blank group and model group (M) received the same amount of saline (1 ml/d). After seven days of continuous administration, 4 mL of blood was collected from the abdominal aorta of each rat from each group. The serum was separated and stored in the refrigerator at −80°C. After collecting the samples, all rats were euthanized by subjecting to the inhalation of excess CO_2_. Two rats from each group were randomly selected, and their colonic tissues were separated for the pathological examination after euthanizing.

### 2.8. Handling of Analytical Samples [13]

The serum samples were subjected to methanol precipitation. Precisely, 0.4 mL of drug-containing serum of the rats from each group was transferred to 1.2 mL of methanol (the volume ratio was 1 : 4), then scrolled for 1 min, and centrifuged at 12000 r/min for 5 min at 4°C. The supernatant was aspirated under dry nitrogen at 40°C. Then, the residue was reconstituted with 200 *μ*L of methanol and sonicated in an ice bath for 10 min. Finally, it was centrifuged at 12000 r/min for 10 min, and the supernatant was used as the test product.

### 2.9. Chromatographic Conditions

Agilent Eclipse XDB C18 chromatographic column (100 mm × 2.1 mm, 3.5 m) was used for separation. The mobile phase was water (A) and methanol (B) (0.1% formic acid) gradient elution, 0–3 min (B: 20%–40%), 3–10 min (B: 40%–54%), 10–25 min (B: 54%), 25–35 min (B: 54%–70%), 35–45 min (B: 70%–80%), 45–50 min (B: 80%), 50–60 min (B: 80%–100%), 70–72 min (B: 100%–20%), and 72–77 min (B: 20%); flow rate, 300 *μ*L/min; column temperature, 40°C; and injection volume, 10 *μ*L. One QC sample was injected into HPLC for instrument calibration among every three test samples, and a total number of 5 QC points were set.

### 2.10. Mass Spectrometry Conditions

ESI source was selected, and scanning was performed in the positive and negative ion modes. The scanning range was 70–1500 *m*/*z*; ion-source gas 1 (GS1): 55 psi; ion-source gas 2 (GS2): 60 psi; CUR: 30 psi; ion-source temperature (TEM): 550°C; ion-source voltage (ISVF): 5500 V/−4500 V; decluster voltage (DP): 100 V; collision energy (CE): 35 V/−35 V; collision energy gain (CES): 15 V/−15 V; and data acquisition mode: IDA.

### 2.11. Analytical Procedure [14, 15]

The high-resolution data were comparatively analyzed. In MarkView 1.3, each set of data was imported, multiple sets of principal component analysis were performed, and the molecular weights and retention times of the substances with large differences (*P* < 0.01 or *P* < 0.05) were derived based on the cluster analysis information. The above information was imported into PeakView 2.0, the molecular compositions of the differential substances were determined by the first-level mass spectrometry, and the molecular structural formula of the substance was determined by the second-level mass spectrometry combined with ChemSpider (https://www.chemspider.com). During qualitative analysis, the top 8000 compounds (*P* < 0.01 or *P* < 0.05) were analyzed individually, and the isotope peaks were selected from the first-level mass spectrum, which was related to the second-level mass spectrum in the order of molecular weight error (<5 ppm), and the molecular formulas of these components were imported into ChemSpider and input in the software in the mol2 format to perform docking.

## 3. Results

### 3.1. Histopathological Observation of Colon

**Figure 1:**
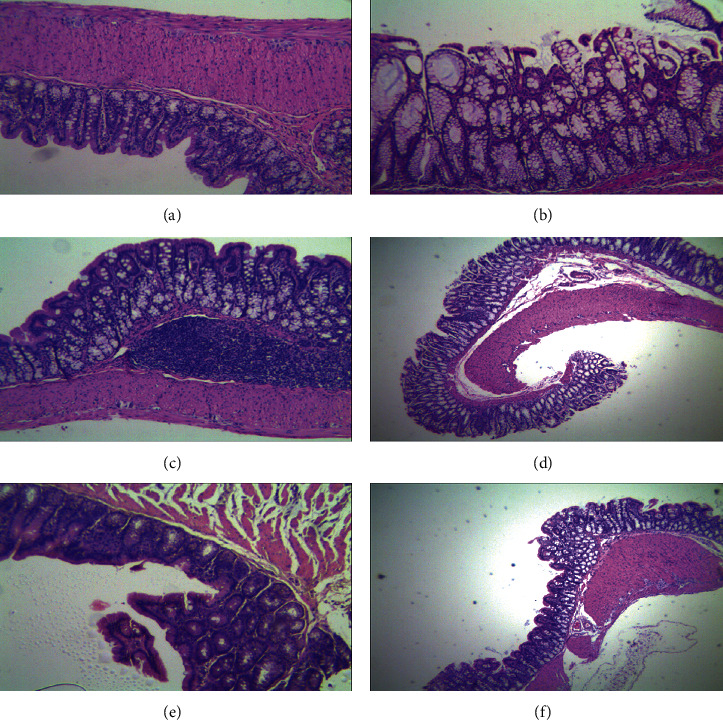
Pathological changes of colonic mucosa (HE, ×40): (a) normal group; (b) M group; (c) MS group; (d) FFKST group; (e) KST group; (f) DYH group.

As shown in Figure 1, the colonic structure of the normal group was clear, and the mucosal epithelium was intact and continuous. Contrarily, the colonic structure was destroyed in the model group (M) with multiple mucosal erosion, punctate necrosis, and hemorrhage. The mucosa in the positive control group (MS) was intact like the mucosa of a newborn. The colons of the compound *Sophora* decoction group (FFKST) and the indirubin group (DYH) were similar to that of the positive control group, while the colons of the matrine and oxymatrine group (KST) were comparable to that of the model group (*M*).

### 3.2. Analysis of Differences in Serum Principal Components of Each Group

**Figure 2:**
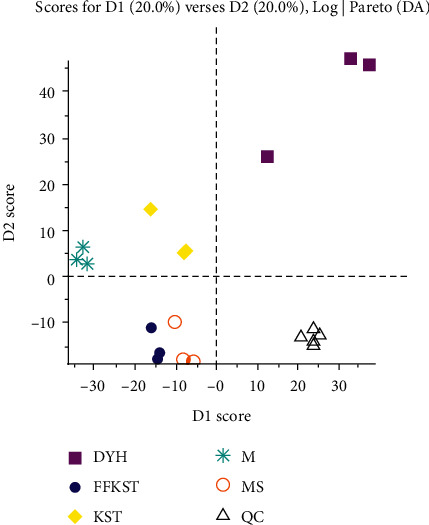
Cluster analysis result obtained by MarkView in negative ion mode (results were output by the software MarkView 1.3).

**Figure 3:**
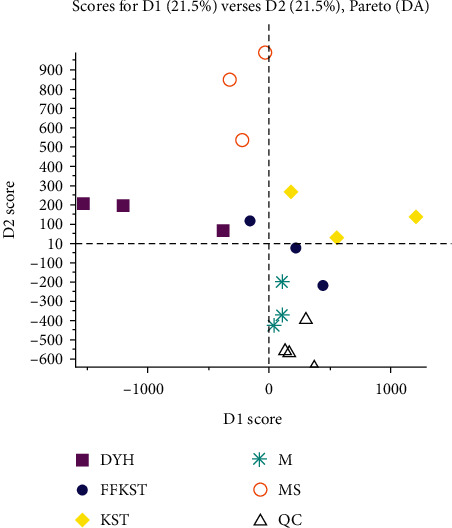
Cluster analysis result obtained by MarkView in positive ion mode (results were output by the software MarkView 1.3).

After all, the above course was processed (including all the samples were processed, they were analyzed in the positive and negative ion modes firstly, and then, the high-resolution data were comparatively analyzed by the software). The processing results obtained by MarkView are shown in Figures 2 and 3. Generally, the absorption, distribution, and metabolism of drugs could inevitably cause changes in the main components of serum. Therefore, the differences in the main components of serum from each group were studied to understand the effects of drugs on endogenous substances. MarkView, special software for metabolomic studies, includes an independent PCA plug-in, which can be used to compare the differences of the main components in two or more groups simultaneously. As shown in figure, the clustering effect of the scatter distribution of different groups in the negative ion mode is obvious. The compound *Sophora* decoction group (FFKST) is spatially close to the positive control group (MS). The matrine and oxymatrine group (KST) is spatially close to the model group (M), but the two of which could still be distinguished. The indirubin group (DYH) is located alone in another space. The results showed that, after the administration of compound *Sophora* decoction, the main components of serum in the model animals were close to those after the positive intervention. Compound *Sophora* decoction played a more consistent role in regulating the serum principal components than the monomer groups. A compound dose of matrine and oxymatrine monomer mixtures did not inflict major changes in the serum principal components of UC-induced rats, and the effects of those mixtures on the serum principal components were almost the same as the model group in terms of category and content without drug intervention. Certainly, differences were also observed between the KST and M groups, so the two mixtures could be distinguished in two dimensions to a very small degree. However, after the indirubin monomers produced a large difference in the serum principal components, the indirubin monomers, separated from the compound, regulated the serum principal components differently than the compound, causing a larger difference, and higher separation was achieved in the clustering results. All of the above was highly consistent with the pathological manifestations of colonic mucosa. In the positive ion mode, different groups failed to distribute the scattered points sufficiently, and the clustering effect was not significant, indicating that the substances, enriched in each group, overlapped in the positive ion mode. In the positive and negative modes, the five samples in the quality control group (QC) were closely placed, and the clustering effect was obvious, indicating that the results were stable and reproducible.

### 3.3. Identification of Differential Substances [16, 17]

**Figure 4:**
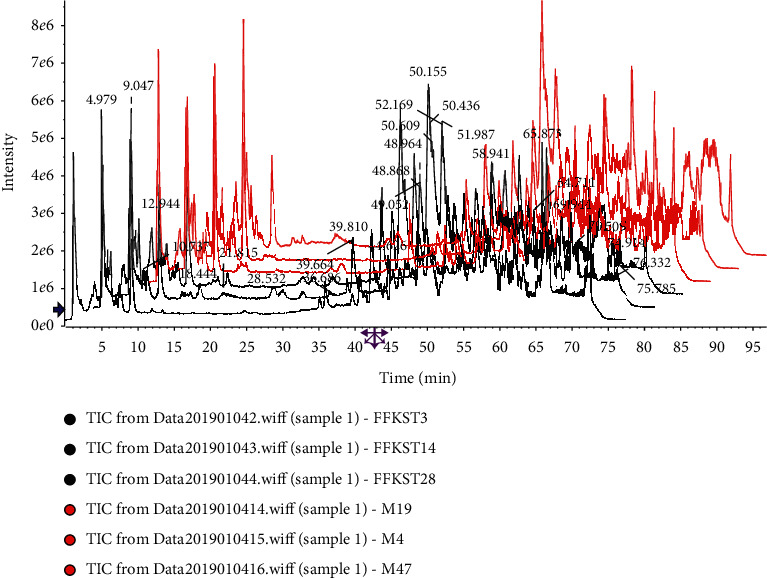
Superposition diagram of the total ion flow of FFKST and M in the negative ion mode.

**Figure 5:**
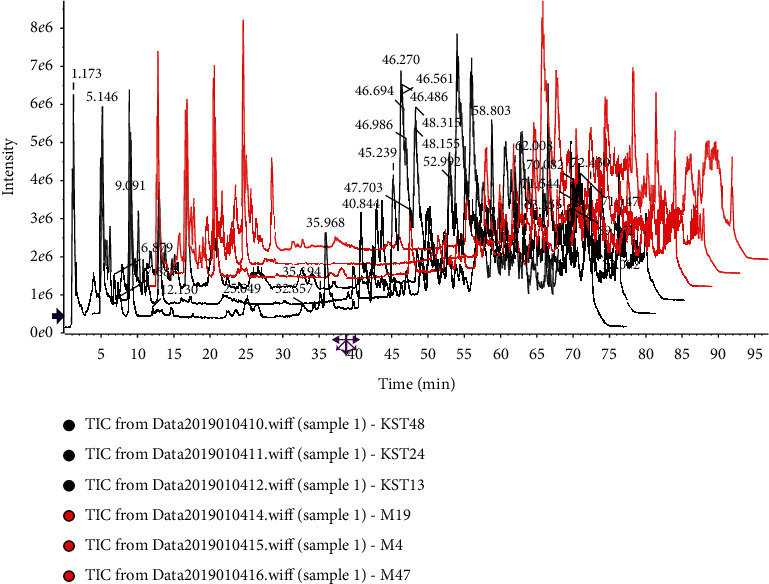
Superposition diagram of total ion flow of KST and M in the negative ion mode.

**Figure 6:**
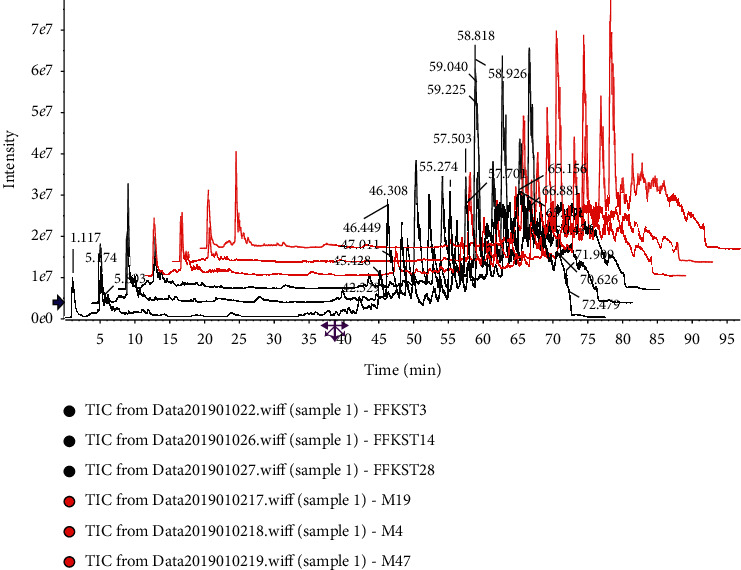
Superposition diagram of total ion flow of FFKST and M in the positive ion mode.

**Figure 7:**
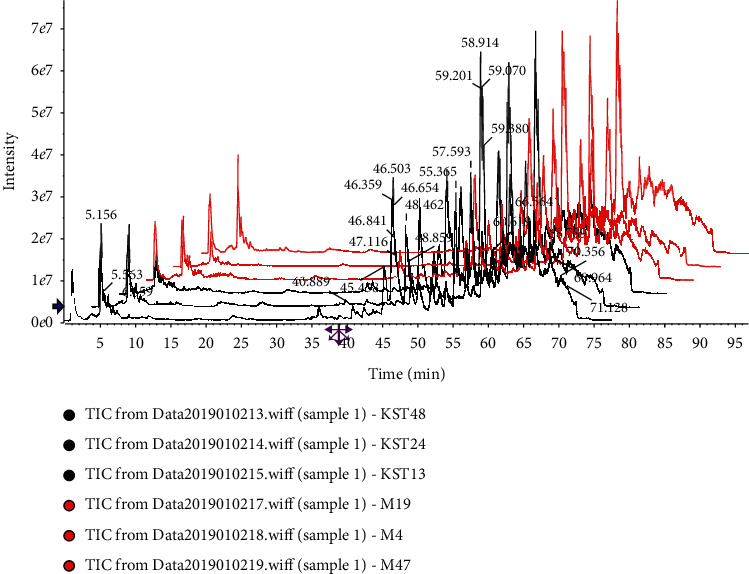
Superposition diagram of total ion flow of KST and M in the positive ion mode.

According to the analytical procedure, the top 8000 compounds with the most significant differences were found in the compound *Sophora* decoction group (FFKST) and the matrine and oxymatrine group (KST) through pairwise comparisons to the model group (M). The superposition maps of total ion flow between compound *Sophora* decoction group (FFKST) and the model group (M) and matrine and oxymatrine group (KST) and the model group (M), are shown in Figures 4–7, which reflect a visual difference between the target group and the model group. The identification results are shown in Tables 1–4. In the negative ion mode, 26 differential compounds were identified in FFKST compared to M, and 18 differential compounds were identified in KST compared to M. In the positive ion mode, 11 and 7 differential compounds were identified in FFKST and KST compared to the model group (M), respectively. Due to the large difference in the related substances produced by the action of the indirubin group (DYH) and the lack of related intersection of different products, the specifically different substances in DYH were not listed in this study. From the results of the analysis, the main discovery under the negative ion was the change in fatty acids and bile acids. In our previous study, we found that the metabolism of various unsaturated fatty acids, such as arachidonic acid and docosatrienoic acid, was abnormal in UC rats. Compound *Sophora* decoction could significantly regulate the metabolism of those compounds in UC mice and sufficiently reduce the inflammatory response and promote the repair of ulcer foci, which also fully demonstrated the integrated regulatory effect of traditional Chinese medicine. Matrine and oxymatrine possess antioxidant activity, and they can regulate blood lipids and protect liver cells [18], which could also inflict changes in fatty acids and bile acids in the body.

**Figure 8:**
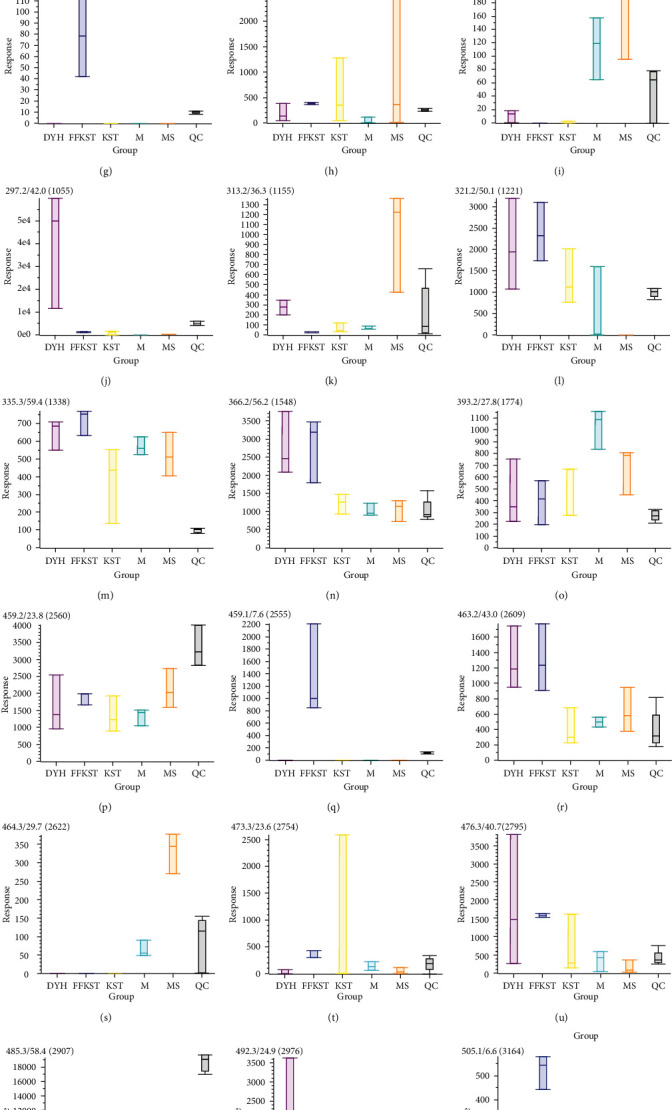
The response signals of 26 differential compounds in FFKST compared to M in each group (*P* < 0.01) in the negative ion mode (results were output by the software MarkView 1.3).

**Figure 9:**
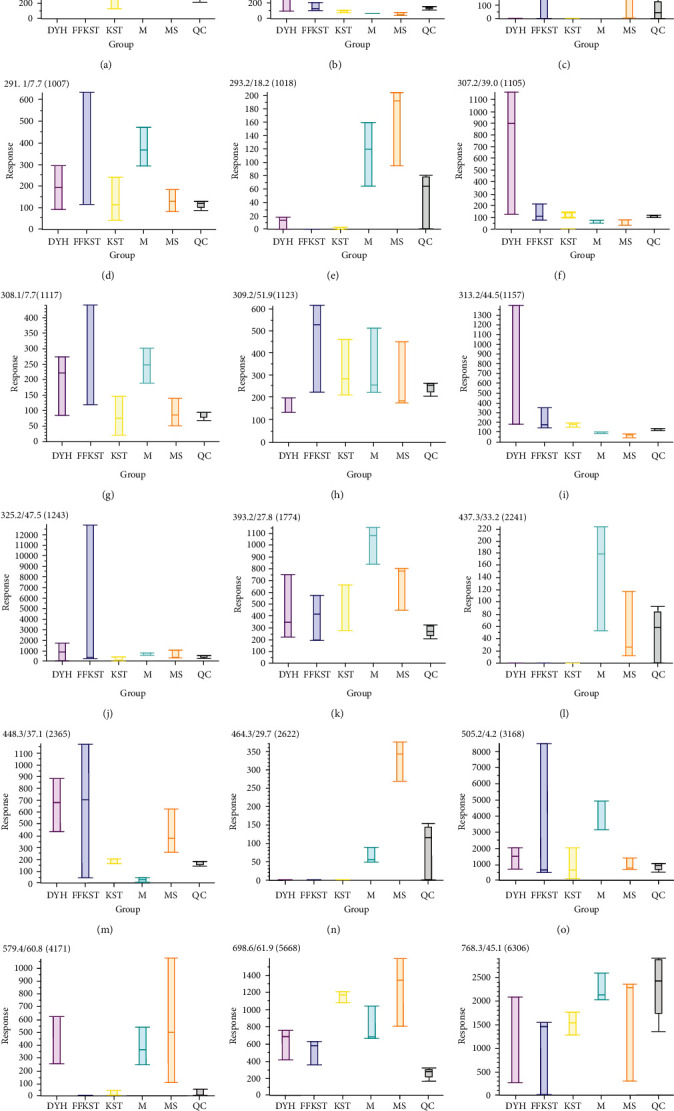
The response signals of 18 differential compounds in KST compared to M in each group (*P* < 0.01) in the negative ion mode (results were output by the software MarkView 1.3).

**Figure 10:**
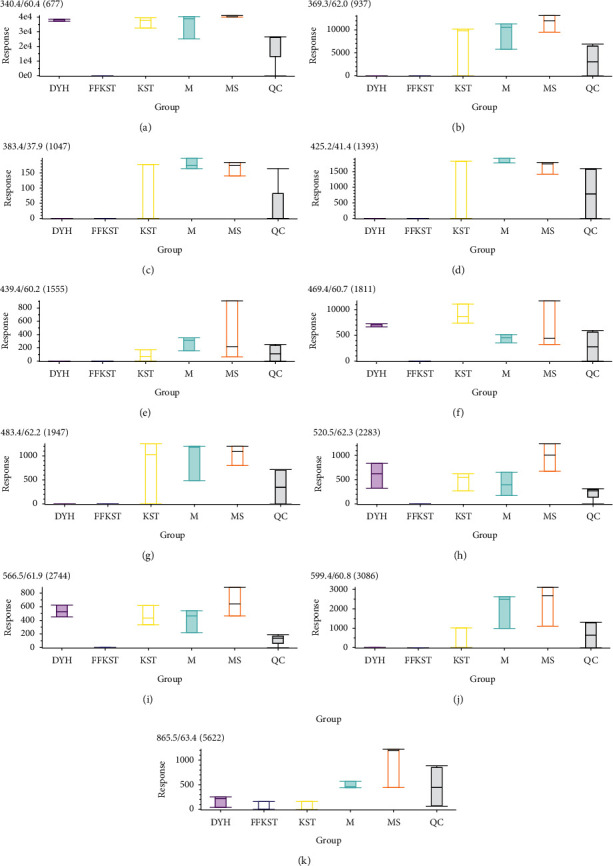
The response signals of 11 differential compounds in FFKST compared to M in each group (*P* < 0.01) in the positive ion mode (results were output by the software MarkView 1.3).

**Figure 11:**
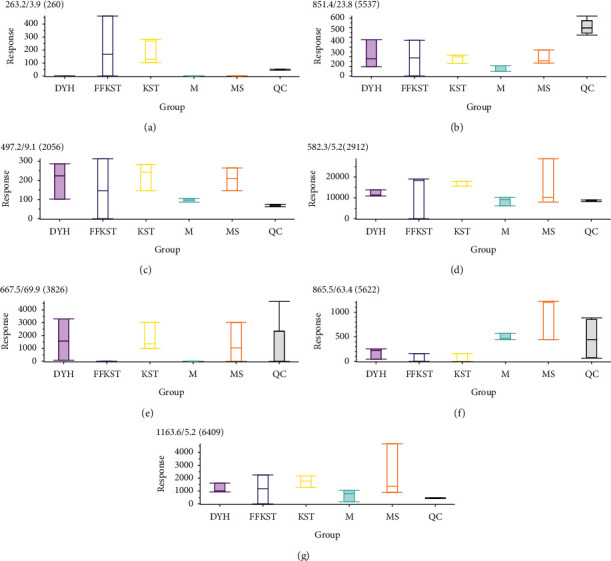
The response signals of 71 differential compounds in KST compared to M in each group (*P* < 0.01) in the positive ion mode (results were output by the software MarkView 1.3).

The main discovery in the positive ion mode was the change in the small peptides. Because a small peptide is originated from different proteins and it is not clear at this stage which proteins are the breakdown products, it is less significant in biological perspectives. In contrast, the changes in the fatty acids and bile acids in the negative ion mode were more consistent with our expectations. Figures 8 and 9 show the response signals of the 26 and 18 differential compounds in each group in negative ion mode, respectively (*P* < 0.01). However, Figures 10 and 11 show the response signal of the 11 and 7 differential compounds in each group in the positive ion mode, respectively (*P* < 0.01).

## 4. Discussion and Conclusion

The intestinal inflammatory damage and the pathological process of UC are closely related to the oxidative stress. Excessive oxygen radicals are involved in different defense mechanisms, including sterilization. Also, it often causes damage to healthy tissues. When the antioxidant system weakens or oxygen radicals are overproduced, oxygen-free radicals can cause damage to surrounding tissues, denature the protein molecules, inactivate enzymes, and even cause cell-death by promoting inflammatory exudation and edema. Excessive free radicals in the intestinal mucosa of UC patients could covalently combine with the multichain unsaturated fatty acids on the cell membrane, triggering a chain reaction of lipid peroxidation [19, 20]. Matrine and oxymatrine can ameliorate the oxidative stress in cells by reducing the absorption of the free fatty acids, enhancing the oxidative utilization of the free fatty acids, enhancing the activity of antioxidant enzymes in cells, and reducing lipid peroxidation [21, 22]. Due to the complex pathogenesis of ulcerative colitis, although the inhibition of excessive antioxidant stress response could provide some limited relief, it cannot produce a significant therapeutic effect.

Compound *Sophora* decoction effectively treats UC and prevents its recurrence. Modern pharmacological studies have shown that *Radix Sophora flavescens* decoction has antibacterial, anti-inflammatory, and leukocyte-increasing effects. *Sanguisorba* decoction could significantly shorten the bleeding and coagulation time, decrease the permeability of capillaries, reduce exudation, minimize tissue edema, form a protective film on the wound surface, promote the healing of the wound, and exert an antibacterial effect. *Indigo naturalis* decoction has a broad-spectrum antibacterial effect. *Bletilla striata* decoction contains colloid components, which could significantly shorten the bleeding and coagulation time, protect gastrointestinal mucosa, promote the formation of granulation tissue, and also exert an antibacterial effect. Therefore, each medicinal component of the compound *Sophora* decoction can synthetically coordinate the function. It can heal the wound and ulcer of the intestinal mucosa, repair the intestinal mucosal barrier, reduce the inflammatory response of the intestinal tract, and improve the immune function of the body. Many investigations have confirmed that the compound *Sophora* decoction can improve the immune function in UC and reduce colonic mucosal IkB-ɑ and NF-kB expression, thereby inhibiting TNF-ɑ and IL-6 expressions. Simultaneously, by inhibiting the activity of STAT6 and disrupting the balance of Th1/Th2 cells, it could improve the inflammatory state in UC and achieve a better therapeutic effect against UC [23–25]. Unlike the monomers, the compound acts through the multipathway and multitarget mechanism. This hypothesis was also confirmed by the difference analysis results of this experiment. By comparing the differences of the serum principal components in the administered groups, we found that the FFKST group could exhibit enhanced synthesis of the serum principal components. However, the mixture of matrine and oxymatrine monomers did not cause the same changes in the serum principal components at the same dose.

In this study, the serum principal components of the target group were compared to the model group for detecting the substances with significant differences and accurately identify the difference in the serum principal components with or without drug intervention. Figures 8–11 show the differences in the response signals of each substance from each group in Tables 1–4. We checked horizontally at the vertical positions of each group; an intersection denotes no significant difference between the groups. Significant differences among the groups are indicated by the absence of any intersection with complete staggering at the vertical level. In the negative ion mode, 26 different compounds were detected in the compound *Sophora* decoction group (FFKST) compared to the model group (M), and five of them were solely present in FFKST with the retention times of 2.0, 3.4, 4.9, 6.6, and 7.6, which were metabolic intermediates. The specific metabolic pathways and pharmacochemical actions should be further studied. Five compounds from the 26 with retention times of 2.7, 43.0, 50.1, 56.2, and 59.4 can also be detected in KST, which were endogenous binders. However, the content difference was significant (*P* < 0.01). Although the type of substance was the same, different contents of the same substances could produce different therapeutic effects. The above results are consistent with the results of matrine and oxymatrine group (KST) in the negative ion mode. In the model group, the components with the retention times of 18.2 and 29.7 were identified as gingerol and glycolic acid. They have anti-inflammatory and analgesic activity. In the absence of drug intervention, the body produces specific immunity, stimulates the synthesis of endogenous active substances, and fights inflammation. However, an organism cannot self-heal when the immunity is low. Indirubin promotes the expression of compact junction proteins, regulates the permeability of intestinal epithelial cells, and reduces the passage of bacteria, endotoxins, and macromolecules through the mucosal barrier into the submucosa. Water molecules and ions pass through the hole where the cord is joined. A charge selective barrier between the intestinal cavity and intestinal mucosa can affect the colonic epithelial cell proliferation/apoptosis and cell migration/repair and regulate the local immune disorders to achieve the therapeutic effect against UC [26, 27]. Although the components in DYH were not researched thoroughly due to the lack of correlation in different principal component substances with other groups, Figures 8–11 can still provide information about the corresponding components in DYH, giving a feedback of differences in both quality and quantity.

In the positive ion mode, most of the 11 differential compounds were small peptides (product of endogenous protein decomposition), which were identified in the model group but were absent in the FFKST group. This study directs further exploration of the pathological mechanism of small peptides. Most of the 7 differential compounds characterized in the comparison between the KST and M groups exhibited signal responses in FFKST and MS, once again proving that the traditional Chinese medicine compound is more comprehensive than monomers. Noteworthy, the differential compounds, we studied, were both endogenous and exogenous. It does not contain the exogenous ingredients, such as matrine and oxymatrine or indirubin, which were administered in the KST and DYH but not in M (which may produce a significant difference in expectation). These compounds could be detected in the blood in the positive ion mode; however, their content was very low. According to the software screening results, the difference compounds were the first 8000 substances with a *P* value ranging from small to large (*P* < 0.01 or *P* < 0.05). As content of these compounds was very low and negligible, they were not included in the results.

## Figures and Tables

**Table 1:** Details of the differential compounds between FFKST and M in the negative ion mode.

Rt (min)	Compounds	Formula	[M −H]^experimental^	[M − H]^calculated^	Error (ppm)	MS/MS
2.71	Unknown	C_9_H_10_O_3_	165.0559	165.0557	1.10	121, 96
3.23	4-Methoxybenzenesulfonic acid	C_7_H_8_O_4_S	187.0072	187.0071	0.80	107, 79
4.87	2-(4-Hydroxybutyl)succinic acid	C_8_H_14_O_5_	189.0770	189.0768	0.80	145, 129, 127, 99
10.65	*N*-Boc-4-piperidineethanol	C_12_H_23_NO_3_	228.1607	228.1605	0.80	211, 167
3.42	4-Methylcatechol-O,O-diacetic acid	C_11_H_12_O_6_	239.0559	239.0561	−0.90	179, 177, 149
50.09	Palmitelaidic acid	C_16_H_30_O_2_	253.2169	253.2173	−1.60	235
2.01	Piscidic acid	C_11_H_12_O_7_	255.0505	255.051	−2.10	175, 165, 149, 135, 93
4.20	Benzyl D-glucopyranuronate	C_13_H_16_O_7_	283.0820	283.0823	−1.20	107, 87
18.17	[6]-Gingerol	C_17_H_26_O_4_	293.1753	293.1758	−1.80	236, 221, 205, 192, 177
42.04	Ricinoleic acid	C_18_H_34_O_3_	297.2427	297.2435	−2.80	279, 183
36.27	Hexanedioic acid, dihexyl ester	C_18_H_34_O_4_	313.2375	313.2384	−3.00	295, 277, 213, 201, 171, 000
50.09	Unknown	C_15_H_26_N_6_O_2_	321.2040	321.2044	−1.40	253, 235
59.35	Unknown	C_18_H_36_N_6_	335.2926	335.2929	−0.80	130, 97
56.21	Unknown	C_15_H_33_N_3_O_7_	366.2240	366.2246	−1.60	281
27.85	12-[2-(3-Carboxypropoxy)phenoxy]dodecanoic acid	C_22_H_34_O_6_	393.2287	393.2296	−2.30	257, 231
7.62	Unknown	C_17_H_17_N_8_O_6_P	459.0916	459.0936	−4.30	283, 255, 240
23.78	Unknown	C_25_H_32_O_8_	459.2023	459.2024	−0.30	323, 254, 186
43.02	Unknown	C_16_H_33_N_8_O_6_P	463.2189	463.2188	0.20	378, 163, 78
29.71	Glycocholic acid	C_26_H_43_NO_6_	464.3017	464.3018	−0.10	402, 74
23.57	Unknown	C_22_H_38_N_2_O_9_	473.2503	473.2505	−0.30	405, 361
40.70	Unknown	C_21_H_39_N_3_O_9_	476.2610	476.2614	−0.70	391, 251
58.36	3,6,9,12,15-Pentaoxaheptacos-1-yl-hydrogen sulfate	C_22_H_46_O_9_S	485.2792	485.279	0.50	469, 421, 280
24.86	Unknown	C_23_H_44_NO_6_PS	492.2560	492.2554	1.20	407
6.61	Unknown	C_19_H_10_N_10_O_8_	505.0612	505.061	0.30	329, 314, 298
61.95	Unknown	C_31_H_37_N_3_O_4_	514.2716	514.2711	0.90	485, 470, 116
40.71	Unknown	C_21_H_31_N_13_O_5_	544.2482	544.2498	−3.00	476, 391, 129

**Table 2:** Details of differential compounds between KST and M in the negative ion mode.

Rt (min)	Compounds	Formula	[M − H]^experimental^	[M − H]^calculated^	Error (ppm)	MS/MS
7.68	Butalbital	C_11_H_16_N_2_O_3_	223.109	223.1088	0.8	180, 153
31.13	6-(3-Hydroxypentan-3-yl)-2,2,6-trimethyloxane-3-carboxylic acid	C_14_H_26_O_4_	257.1759	257.1758	0.3	239, 195
57.88	Stearic acid	C_18_H_36_O_2_	283.2641	283.2643	−0.50	265
7.68	3′-Azido-2′,3′-dideoxyguanosine	C_10_H_12_N_8_O_3_	291.0962	291.096	0.8	291, 223, 180
18.24	[6]-Gingerol	C_17_H_26_O_4_	293.1762	293.1758	1.30	236, 221, 205, 192, 177
39.1	Unknown	C_14_H_24_N_6_O_2_	307.1892	307.1888	1.3	259, 239, 102
7.66	2-({5-Chloro-2-[(2-fluorobenzyl)oxy]benzyl}amino)ethanol	C_16_H_17_NO_2_FCl	308.0863	308.0859	1.3	223, 180
51.95	Unknown	C_14_H_26_N_6_O_2_	309.2045	309.2044	0.2	241, 104
44.5	Octadecanedioic acid	C_18_H_34_O_4_	313.2385	313.2384	0.2	295, 251
47.53	2-Dodecylbenzenesulfonic acid	C_18_H_30_O_3_S	325.1843	325.1843	0	293, 183
27.82	12-[2-(3-Carboxypropoxy)phenoxy]dodecanoic acid	C_22_H_34_O_6_	393.2282	393.2283	−0.2	257, 231
37.12	Glycodeoxycholic acid	C_26_H_43_NO_5_	448.3062	448.3068	−1.4	404, 74
29.67	Glycocholic acid	C_26_H_43_NO_6_	464.3013	464.3018	−1	402, 74
4.19	Unknown	C_19_H_34_N_6_O_10_	505.2261	505.2264	3.00	306, 241, 207
60.82	2-Deoxy-4-O-[(2E,4R,6E,8E,10S,11R,12S)-11-hydroxy-2,4,6,8,10,12-hexamethyl-5-oxo-2,6,8-docosatrienoyl]-D-erythro-pentonic acid	C_33_H_56_O_8_	579.3901	579.3902	−0.2	403, 175, 113
46.14	Unknown	C_26_H_48_N_7_O_9_P	632.3177	632.3178	−0.2	504, 279, 112
61.95	Galactosylceramide	C_40_H_77_NO_8_	698.558	698.5576	0.5	536, 426, 290, 179, 89
45.06	Unknown	C_28_H_59_N_3_O_13_P_4_	768.2929	768.2926	0.4	248, 180, 112

**Table 3:** Details of differential compounds between FFKST and *M* in the positive ion mode.

Rt (min)	Compounds	Formula	[M + H]^experimental^	[M + H]^calculated^	Error (ppm)	MS/MS
60.41	Docosanamid	C_22_H_45_NO	340.3565	340.3574	−2.6	102, 88
62.02	(20R)-Cholesta-3,5-dien	C_27_H_44_	369.3501	369.3516	−4	287, 243, 215, 175, 161, 147, 109, 95
37.99	Val-his-ala-gly	C_16_H_26_N_6_O_5_	383.2033	383.2037	−1.2	327, 281, 267
41.45	Propyl-6-O-(2-acetamido-2-deoxy-*β*-D-glucopyranosyl)-2-amino-2-deoxy-*α*-D-glucopyranoside	C_17_H_32_N_2_O_10_	425.2118	425.213	−2.8	355, 281
60.21	4-[(2-Cyanoethyl)amino]-3-hydroxy-2,2-dimethyl-4-oxobutyl-palmitate	C_25_H_46_N_2_O_4_	439.3536	439.353	1.3	407
60.73	3-(1-Hydroxy-2,4-dimethylhexyl)-2-isopropoxy-5,6-dimethoxy-4-pyridinyl diisopropylcarbamate	C_25_H_44_N_2_O_6_	469.3272	469.3272	0	329, 189
62.21	*N*-[(1S,2S,8S,8aS)-7-{(2S)-1-[Bis(2-methoxyethyl)amino]-1-oxo-2-propanyl}-8-hydroxy-1,4a-dimethyldecahydro-2-naphthalenyl]-3,3-dimethylbutanamide	C_27_H_50_N_2_O_5_	483.3782	483.3792	−2.2	453
62.39	*N*-[(9Z)-9-Octadecen-1-yl]-2-oxohexadecanamide	C_34_H_65_NO_2_	520.5056	520.5088	−6.2	282, 264, 252
61.88	(2R,4S,5S,7S)-5-Amino-*N*-butyl-7-{4-[4-(dimethylamino)butoxy]-3-(3-methoxypropoxy)benzyl}-4-hydroxy-2,8-dimethylnonanamide	C_32_H_59_N_3_O_5_	566.4512	566.4527	−2.7	226, 184, 104
60.86	L-Valyl-L-isoleucylglycyl-L-alanyl-*N*-[(2S)-6-amino-1-oxo-2-hexanyl]-L-lysinamide	C_28_H_54_N_8_O_6_	599.4247	599.4239	1.3	300
63.68	L-Lysyl-L-seryl-L-leucyl-N5-(diaminomethylene)-L-ornithyl-L-seryl-L-phenylalanyl-L-lysine	C_39_H_68_N_12_O_10_	865.5265	865.5254	−1.2	780, 721, 597, 575, 145.00

**Table 4:** Details of differential compounds between KST and M in the positive ion mode.

Rt (min)	Compounds	Formula	[M + H]^experimental^	[M + H]^calculated^	Error (ppm)	MS/MS
3.94	3-(4-Hydroxypiperidin-1-yl-methyl)benzimidic acid ethyl ester	C_15_H_22_N_2_O_2_	263.1756	263.1754	0.7	245, 162, 96
23.97	7-(2-Hydroxy-3-isopropoxypropyl)-3-methyl-8-[(2E)-2-(1-phenylethylidene)hydrazino]-3,7-dihydro-1H-purine-2,6-dione	C_20_H_26_N_6_O_4_	415.2096	415.2088	2.3	135, 119, 91
9.1	Tyr-ser-asp-ile	C_22_H_32_N_4_O_9_	497.2262	497.2242	4	427, 357, 337, 271
5.22	Unknown	C_48_H_82_N_12_O_21_	582.2928	582.2932	−0.6	961, 582, 217, 85
5.12	1-Oleoyl-2-(4Z,7Z,10Z,13Z,16Z,19Z-docosahexaenoyl)-sn-glycerol	C_43_H_70_O_5_	667.5266	667.5296	−4.6	383, 341
63.68	L-Lysyl-L-seryl-L-leucyl-N5-(diaminomethylene)-L-ornithyl-L-seryl-L-phenylalanyl-L-lysine	C_39_H_68_N_12_O_10_	865.5265	865.5254	−1.2	780, 721, 597, 575, 145.00
69.89	Unknown	C_44_H_78_N_18_O_19_	1163.576	1163.576	−0.2	1074

## Data Availability

All data, models, or code generated or used during the study are available from the corresponding author by request.

## References

[1] Zuo D., Liu X., Shou Z. (2013). Study on the interactions between transplanted bone marrow-derived mesenchymal stem cells and regulatory T cells for the treatment of experimental colitis. *International Journal of Molecular Medicine*.

[2] Zhang X. M., Shou Z. X., Fan H. (2014). Effect of QRZSLXF on colon tissue repair in rats with ulcerative colitis. *Journal of Traditional Chinese Medicine*.

[3] Zuo D., Tang Q., Fan H. (2015). Modulation of nuclear factor-*κ*B-mediated pro-inflammatory response is associated with exogenous administration of bone marrow-derived mesenchymal stem cells for treatment of experimental colitis. *Molecular Medicine Reports*.

[4] Zhou P. Q., Fan H., Hu H. (2014). Role of DOR-B-arrestin1-Bcl2 signal transduction pathway and intervention effects of oxymatrine in ulcerative colitis. *Huazhong University of Science and Technology*.

[5] Wu S.-T. (2017). *Study on the Effective Components and Mechanism of Compound Sophorae Decoction against Ulcerative Colitis*.

[6] Schrattenholz A., Groebe K., Soskic V. (2010). Systems biology approaches and tools for analysis of interactomes and multi-target drugs. *Methods in Molecular Biology*.

[7] Yi Z., Fan H., Liu X., Tang Q., Zuo D., Yang J. (2015). Adrenomedullin improves intestinal epithelial barrier function by downregulating myosin light chain phosphorylation in ulcerative colitis rats. *Molecular Medicine Reports*.

[8] Wang X. L., Hong Z. C., Wu S. T. (2017). Determination of matrine and oxymatrine in compound sophora decoction by HPLC. *Central South Pharmacy*.

[9] Zhou C. Z., Jiang N., Zhou C. H. (2016). Study on the intervention and mechanism of compound sophora decoction in rats with ulcerative colitis model. *China Pharmacist*.

[10] Hong Z. C., Duan X. Y., Caia Q. (2020). Study of compound sophorae decoction in the treatment of ulcerative colitis by tissue extract metabonomics approach. *Journal of TCM*.

[11] Fan H., Liao Y., Tang Q. (2012). Role of *β*_2_-adrenoceptor-*β*-arrestin2-nuclear factor-*κ*B signal transduction pathway and intervention effects of oxymatrine in ulcerative colitis. *Chinese Journal of Integrative Medicine*.

[12] Fan H., Liu X.-X., Zhang L.-J. (2014). Intervention effects of QRZSLXF, a Chinese medicinal herb recipe, on the DOR-*β*-arrestin1-Bcl2 signal transduction pathway in a rat model of ulcerative colitis. *Journal of Ethnopharmacology*.

[13] He X.-Y., Liu Q.-C., Peng W., Huang Y.-L., Wu C.-J. (2013). Bioactivities and serum pharmacochemistry ofQi-Wei-Xiao-Yan-Tang. *Pharmaceutical Biology*.

[14] Zhu G. T., Wang S. C., Huang Z. J. (2018). Rewiring of the fruit metabolome in tomato breeding. *Cell*.

[15] He Z., Wang Y., Zhang Y., Cheng H., Liu X. (2018). Stereoselective bioaccumulation of chiral PCB 91 in earthworm and its metabolomic and lipidomic responses. *Environmental Pollution*.

[16] Zhang J. K., Wang P., Wei X. (2015). A metabolomics approach for authentication of ophiocordyceps sinensis by liquid chromatography coupled with quadrupole time-of-flight mass spectrometry. *Food Research International*.

[17] Zhang J. K., Yu Q. H., Cheng H. Y. (2018). Metabolomic approach for the authentication of berry fruit juice by liquid chromatography quadrupole time-of-fight mass spectrometry coupled to chemometrics. *Journal of Agricultural & Food Chemistry*.

[18] Huang L. H., Zhong Y. M., Xiong X. H. (2016). The disposition of oxymatrine in the vascularly perfused rat intestine-liver preparation and its metabolism in rat liver microsomes. *Journal of Pharmaceutical Sciences*.

[19] Deng S., Tang Q., Duan X. (2019). Uncovering the anticancer mechanism of compound sophorae decoction against ulcerative colitis-related colorectal cancer in mice. *Evidence-Based Complementary and Alternative Medicine*.

[20] Xu M., Duan X.-Y., Chen Q.-Y. (2019). Effect of compound sophorae decoction on dextran sodium sulfate (DSS)-induced colitis in mice by regulating Th17/Treg cell balance. *Biomedicine & Pharmacotherapy*.

[21] Zou Z., Zuo D., Yang J., Fan H. (2016). The ANXA1 released from intestinal epithelial cells alleviate DSS-induced colitis by improving NKG2A expression of natural killer cells. *Biochemical and Biophysical Research Communications*.

[22] Liu W, Baker S., Baker R., Ziu L. (2015). Antioxidant mechanisms in nonalcoholic fatty liver disease. *Current Drug Targets*.

[23] ZhuBaker D. E., Friedman J. E. (2014). Developmental origins of nonalcoholic fatty liver disease. *Pediatric Research*.

[24] Zhou P.-Q., Fan H., Hu H. (2014). Role of DOR-P-arrestin1-Bcl2 signal transduction pathway and intervention effects of oxymatrine in ulcerative colitis. *Journal of Huazhong University of Science and Technology [Medical Sciences]*.

[25] Chen Q., Duan X., Xu M. (2020). BMSC-EVs regulate Th17 cell differentiation in UC via H3K27me3. *Molecular Immunology*.

[26] Inagaki-Ohara K., Dewi F. N., Hisaeda H. (2006). Intestinal intraepithelial lymphocytes sustain the epithelial barrier function against eimeria vermiformis infection. *Infection and Immunity*.

[27] Gunzel D., Florian P., Richter J. F. (2006). Restitution of single cell defects in the mouse colon epithelium differs from that of cultured cells. *American Journal of Physiology-Regulatory, Integrative and Comparative Physiology*.

